# Heterogeneity in Measures of Illness among Patients with Myalgic Encephalomyelitis/Chronic Fatigue Syndrome Is Not Explained by Clinical Practice: A Study in Seven U.S. Specialty Clinics

**DOI:** 10.3390/jcm13051369

**Published:** 2024-02-28

**Authors:** Elizabeth R. Unger, Jin-Mann S. Lin, Yang Chen, Monica E. Cornelius, Britany Helton, Anindita N. Issa, Jeanne Bertolli, Nancy G. Klimas, Elizabeth G. Balbin, Lucinda Bateman, Charles W. Lapp, Wendy Springs, Richard N. Podell, Trisha Fitzpatrick, Daniel L. Peterson, C. Gunnar Gottschalk, Benjamin H. Natelson, Michelle Blate, Andreas M. Kogelnik, Catrina C. Phan, on behalf of the MCAM Study Group

**Affiliations:** 1 Division of High-Consequence Pathogens and Pathology, Centers for Disease Control and Prevention (CDC), Atlanta, GA 30329-4027, USA; dwe3@cdc.gov (J.-M.S.L.); njl3@cdc.gov (Y.C.); yex8@cdc.gov (M.E.C.); kux0@cdc.gov (B.H.); lxo4@cdc.gov (A.N.I.); jub7@cdc.gov (J.B.); 2 Institute for Neuro Immune Medicine, Nova Southeastern University, Fort Lauderdale, FL 33314, USA; nklimas@nova.edu (N.G.K.); ebalbin@med.miami.edu (E.G.B.); 3 VA Medical Center, Geriatric Research and Education Clinical Center, Miami, FL 33125, USA; 4 Bateman Horne Center, Salt Lake City, UT 84102, USA; 5 Hunter-Hopkins Center, Charlotte, NC 28226, USA; cwlapp@drlapp.net (C.W.L.); wendy@drlapp.net (W.S.); 6 Richard N. Podell Medical, Summit, NJ 07901, USA; 7 Sierra Internal Medicine, Incline Village, NV 89451, USA; dpeterson@sierrainternalmed.com (D.L.P.); ggottschalk@simmaron.com (C.G.G.); 8 Department of Neurology, Mount Sinai Beth Israel, New York, NY 10029, USA; benjamin.natelson@mountsinai.org (B.H.N.); michelle.blate@mountsinai.org (M.B.); 9 Open Medicine Clinic, Mountain View, CA 94040, USA; andy@y-dna.com (A.M.K.);

**Keywords:** multi-site study, site difference, patient characteristics, heterogeneity, common data elements (CDEs), myalgic encephalomyelitis/chronic fatigue syndrome (ME/CFS)

## Abstract

**Background:** One of the goals of the Multi-site Clinical Assessment of Myalgic Encephalomyelitis/Chronic Fatigue Syndrome (MCAM) study was to evaluate whether clinicians experienced in diagnosing and caring for patients with myalgic encephalomyelitis/chronic fatigue syndrome (ME/CFS) recognized the same clinical entity. **Methods:** We enrolled participants from seven specialty clinics in the United States. We used baseline data (n = 465) on standardized questions measuring general clinical characteristics, functional impairment, post-exertional malaise, fatigue, sleep, neurocognitive/autonomic symptoms, pain, and other symptoms to evaluate whether patient characteristics differed by clinic. **Results:** We found few statistically significant and no clinically significant differences between clinics in their patients’ standardized measures of ME/CFS symptoms and function. Strikingly, patients in each clinic sample and overall showed a wide distribution in all scores and measures. **Conclusions:** Illness heterogeneity may be an inherent feature of ME/CFS. Presenting research data in scatter plots or histograms will help clarify the challenge. Relying on case–control study designs without subgrouping or stratification of ME/CFS illness characteristics may limit the reproducibility of research findings and could obscure underlying mechanisms.

## 1. Background/Introduction

Myalgic encephalomyelitis/chronic fatigue syndrome (ME/CFS) is recognized to be a significant illness that impairs the lives of those affected and their families. The economic burden of the illness in the U.S. is estimated to be USD 9–14 billion annually in direct medical costs and an additional USD 9–37 billion annually in lost productivity [1,2]. Patients with ME/CFS experience a wide range of symptoms, most characteristically significantly decreased function associated with severe fatigue, post-exertional malaise, unrefreshing sleep, cognitive impairment, and orthostatic intolerance. However, chronic widespread pain, allergies, sensitivity to light and sound, chemical and food sensitivities, headaches, and other symptoms are common. The pathogenesis of this biologic illness remains a mystery despite decades of research. While a wide variety of objective differences between patients with ME/CFS and healthy controls have been reported, none are specific and sensitive enough to be used in diagnosis [3]. 

In the absence of a diagnostic test, recognizing ME/CFS requires reliance on patient-reported characteristic symptoms and clinical acumen to identify and treat any other illnesses that may contribute to the problems experienced by the patient. A variety of research and clinical case definitions have been in use, and clinicians world-wide recognize the clinical entity [4]. While the definitions have consensus on core features, differences in the numbers of required symptoms and in the use of exclusionary conditions contribute to variations in diagnosis. Further, the case definitions do not include guidance on how symptom-based criteria are fulfilled, and different approaches to applying the same case definition can affect diagnosis [5]. The 2015 Institute of Medicine report on ME/CFS recommended a streamlined clinical case definition to aid healthcare providers in recognizing the illness [6]. Nonetheless, case ascertainment for research purposes remains controversial, and differences in study findings may arise from the lack of defined reproducible measures and criteria for each element of a case definition. Currently, many studies rely on a combination of clinical expertise and one or more case definitions. Further, as many studies rely on patients recruited from a single specialty clinic, differences in referral patterns and clinical practices could affect patient characteristics. 

One of the goals of the Multi-site Clinical Assessment of Myalgic Encephalomyelitis/Chronic Fatigue Syndrome (MCAM) study was to evaluate whether clinicians experienced in diagnosing and caring for patients with ME/CFS recognized the same clinical entity. This question is important for both research and clinical practice. Are there clinic-based differences resulting in the identification of patients that differ in important ways between clinics? Such differences could result in challenges replicating study findings and contribute to clinical responses to therapies. We used standardized data collected on key illness dimensions of patients with ME/CFS enrolled from seven specialty clinics in the U.S. to investigate whether these patient characteristics differ between clinics. 

## 2. Methods

We have published details of the study design and methods of the source study, MCAM [7]. Briefly, expert clinicians from 7 specialty clinics in the U.S. enrolled patients who, in their clinical opinion, met criteria for ME/CFS and were between the ages of 18 and 70 years at the time of enrollment. Clinical sites were designated by letters (A–G). Each clinical site completed a form to provide information describing their clinical practice [Form included in Supplemental Material S1].

Clinic personnel systematically abstracted socio-demographic data and presentation of illness from medical records. Patients completed a battery of standardized questionnaires/forms to measure ME/CFS symptoms and illness domains (Table 1). Although the MCAM study was initiated prior to the publication of the first set of Common Data Elements (CDEs) for ME/CFS, the study collected many of these data elements via their recommended assessment tools [8].

We have published the overall socio-demographic characteristics of the initial study sample (n = 471) from baseline enrollment between January 2012 and May 2013 [7]. Six of those enrolled were later confirmed to be age-ineligible and removed from further analysis; thus, the current analysis includes data from 465 ME/CFS patients. Patient characteristics are examined by site (A through G) as well as overall. 

In addition to examining differences in patient characteristics by site of enrollment, we sought to estimate whether patients met the most frequently used case definitions of ME/CFS or CFS. We operationalized the application of each case definition by using standardized measures and scoring thresholds for each required element of the definition. The 1994 research case definition of CFS [9] was evaluated using items from the CDC Symptom Inventory, SF-36, and MFI-20 [5]. The 2003 Canadian case definition for ME/CFS [10] was evaluated using items from the DePaul Symptom Questionnaire, as previously reported [11]. The 2015 IOM clinical case definition [6] was evaluated using items from the CDC Symptom Inventory and the DePaul Symptom Questionnaire. Details of the algorithms are included in Supplemental Materials (S2). 

Descriptive statistics were calculated by site. Means and standard deviations were calculated for continuous variables, and proportions were calculated for categorical variables. Variation in patient characteristics, overall and by site, were examined using a combination of graphical (e.g., bar graphs, boxplots) and numerical (e.g., general linear models for continuous variables and chi-square tests for categorical variables) methods. Bonferroni-corrected p-values were calculated for the multiple group comparisons on outcome measures of interest.

## 3. Results

### 3.1. Clinical Practice Characteristics of Study Sites

Each of the seven expert clinicians established clinics to provide care to patients with ME/CFS. Most of them also cared for patients with fibromyalgia, fatigue, and sleep and/or pain disorders. The primary physicians at each clinical site had between 15 and 45 years’ experience with diagnosing and caring for patients with ME/CFS, and their clinical practices had been active for 5–40 years. The board certifications of study physicians varied and included internal medicine, environmental medicine, infectious disease, immunology, hematology, neurology, and pediatrics. About half of the clinical sites required payment from their patients rather than relying on insurance. While over 75% of clinics indicated use of an electronic health record (EHR) system, this was limited to billing and payment for the majority. Most clinics did not use an EHR system until 2012, the first enrollment year of the MCAM study. Three sites were solo practices, two were small group, one was academic, and one was hospital-based. Clinic staff included nurses and/or nurse practitioners. One clinic had a physician assistant. The number of patients seen at each clinical site ranged from 5 to 70 per week. Six clinics accepted new patients, and waiting lists at four clinics ranged between 40 and 700 patients. Clinics varied in their location (four metropolitan, two urban, and one rural), ease of accessibility (public transportation or on-site parking available at all sites), and other important characteristics (time spent with patients and charges for the initial and follow-up visits). 

### 3.2. Description of Study Sample

#### 3.2.1. Socio-Demographic Characteristics

**Figure 1:**
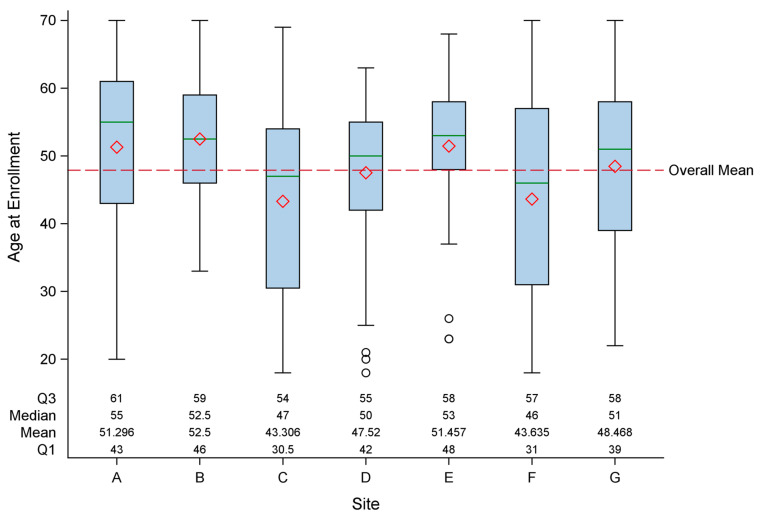
Distribution of age at enrollment by site (A through G) and overall mean. The boxplots display the five-number summary: minimum, first quartile (Q1), median, third quartile (Q3), and maximum. The central rectangle spans from the first quartile to the third quartile (the interquartile range), a green segment inside the rectangle shows the median, the red diamond shows the mean, and the vertical lines (sometimes referred to as whiskers) are extended to the extrema of the distribution in the data set.

As shown in Table 2, other than employment, each of these patient characteristics differed by site according to statistical measures; however, there was also significant variation for each measure within individual clinics. For example, overall, the mean age at enrollment was 47.9 years (standard deviation (SD) = 12.6), and the range in the mean age by site was 43.3 to 52.5 years. As shown in Figure 1, there was extensive overlap in the age distributions between sites (overall range <20 to 70 years). Females predominated at each site, with an overall female-to-male ratio of 2.9 (range of 1.6 to 5.6 across sites). Patients were predominantly white, comprising 94.4% of the study sample (range of 84.4% to 100% across sites). More than half were married or in a committed relationship, but the proportion ranged from 40.8% to 73.3% across sites. Overall, about 75% were unemployed (range of 64.3% to 89.1% across sites). The majority were insured (94.3%) and at least college graduates (76.8%), but the insured rate ranged from 87% to 100%. The proportion attaining a college degree or higher ranged from 58.9% to 85.9%.

#### 3.2.2. General Clinical Characteristics

**Figure 2:**
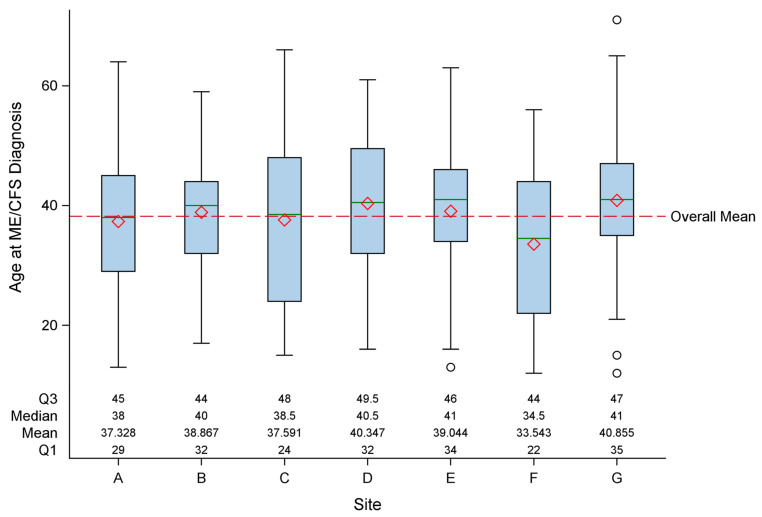
Distribution of age at diagnosis by site (A through G). The boxplots display the five-number summary: minimum, first quartile (Q1), median, third quartile (Q3), and maximum. The central rectangle spans from the first quartile to the third quartile (the interquartile range), a green segment inside the rectangle shows the median, the red diamond shows the mean, and the vertical lines (sometimes referred to as whiskers) are extended to the extrema of the distribution in the data set.

Table 3 presents the general characteristics of the patients overall and by site, including age at diagnosis, mode of onset, duration of fatigue, Body Mass Index (BMI), and number of medications. Each of these characteristics differed by site according to statistical measures. Overall, the mean age at diagnosis was 38.3 years (SD = 12.6), whereas by site, the mean age at diagnosis ranged from 33.5 to 40.9 years. As shown in Figure 2, the distribution of ages at diagnosis by clinic and overall is broad and shows substantial overlap. Sudden illness onset was reported by 65.4% of patients overall, and this varied significantly by clinic, ranging from 49.3% to 75.4%. In all but one clinic, sudden onset was more common than gradual. The mean duration of fatigue ranged from 9.4 to 17.9 years across sites. We used the duration of fatigue as a surrogate for the duration of ME/CFS, as not all symptoms may appear at the same time. On a clinic basis, the mean age at enrollment and the duration of fatigue showed a modest correlation (R^2^ = 0.74). The mean BMI was in the overweight range for all clinics (range of 24.3–29.2), overall, 26.6 (SD = 6.3). The mean number of medications was 5.9 overall, with variation by site (range of means of 4.1–8.2). 

### 3.3. Description of Illness

#### 3.3.1. Functional Impairment

Table 4 shows the patients’ measures of functional impairment by site and overall. These measures show no statistical differences among the clinics. As presented previously for the overall group [7], the mean scores for Role Physical and Vitality were lowest (worst functioning) for patients at each site, and the mean scores for Mental Health and Role Emotional were highest (least impaired) among SF-36 subscales. 

Hours of vertical activity is the patient-reported average number of hours per day that they spent with their feet on the floor. This encompasses sitting, standing, and walking activities. The median of patients’ vertical hours by site ranged from 6.3 to 8.0, with an overall mean of 7.5 h. To help put this into context, this is half of that reported by healthy controls (14 h) [12].

Exercise was evaluated using the response to the level of physical activity questions (medical history form) and the question about how many times in a typical week participants would exercise for more than 15 minutes in their leisure time. Strenuous exercise was described as “heart beating rapidly” (examples: soccer, jogging). Moderate exercise was described as “not exhausting” (examples: lifting weights, fast walking, folk dancing). Mild exercise was described as “minimal effort” (examples: yoga, golf, bowling). The highest level of exercise engaged at least once per week for more than 15 min was captured. Overall, more than half (73.6%) of all patients reported engaging in mild exercise, with a range across sites of 65.3% to 84.1%. Fewer patients (29.5% overall, 23.3% to 36.4% across sites) reported engaging in moderate exercise, while even fewer reported strenuous exercise at least once a week (7.8% overall, 4.1% to 11.6% across sites). 

The CDC Health-related Quality of Life (HRQoL) asks patients to record the number of days during the past 30 days when their physical health (includes physical illness and injury) was not good. Overall, the mean number of physically unhealthy days experienced by patients in the past 30 days was 23.2 days (SD = 9.5), and by site, the means ranged from 20.5 to 25.1 days. 

#### 3.3.2. Measures of Post-Exertional Malaise (PEM)

We evaluated PEM using medical record review (Illness Abstraction Form [IAF]) as well as portions of two different questionnaires, the CDC SI (PEM question) and DePaul Symptom Questionnaire (items 14–18). The CDC SI and DSQ provided item scores and a determination of PEM presence. The results are shown in Table 5 by site and overall. None of these measures differed significantly by site. 

The IAF was completed by clinic staff based on the review of all available medical records for an indication that PEM, with illness relapse of at least 24 h duration, was experienced by the patient for at least 6 months at any point in their illness. PEM was recorded as present based on the IAF if these criteria were met. Overall, PEM could be identified by the IAF in 87.5% of patients, with a range by clinic of 83.8% to 90.7%. 

Based on the CDC SI, PEM was recorded as present if the symptom lasted at least 6 months and was reported during the past month. Overall, the CDC SI identified the presence of PEM in 94.7% of patients with a mean score of 11.7 (SD = 4.8). The mean score by site ranged from 10.8 (SD = 5.1) to 12.6 (SD = 3.3), and PEM presence varied from 90.3% to 100%. 

Based on the DSQ, PEM was determined to be present if any of the five DSQ items had values for both frequency and severity ≥ 2. Overall, the mean for each of the five PEM items ranged from a high of 9.1 (SD = 5.4) for item 17 (tired after minimal exercise) to a low of 6.5 (SD = 5.1) for item 16 (mentally tired after slightest effort). The mean PEM item scores by site but did not differ significantly. Overall, the DSQ identified PEM in 94.0% of patients, and the range by site was 89.1% to 97.4%. 

The three approaches to evaluating PEM were compared in the 461 participants with enough information. Overall, PEM was identified by all three methods in 81% (371/461) of participants, and only 6 (1.3%) failed to have PEM documented with at least one method. Concordance was highest between the DSQ and CDC-SI (92.4%, 426/461) and lowest between the IAF and DSQ (83.5%, 385/461).

#### 3.3.3. Measures of Fatigue

Table 6 shows the mean scores on the MFI-20 subscales and the mean T-score of the PROMIS Fatigue Short Form 7a (PROMIS F-SF) by site and overall. Most of these measures show no statistically significant difference by site. The exceptions are two of the MFI-20 subscales, Physical Fatigue and Reduced Activity. However, the mean difference in these subscale scores across sites is less than the two-point minimal clinically important difference for these measures. 

#### 3.3.4. Measures of Sleep

Table 7 shows the mean scores of sleep measures overall and by site. The measures include unrefreshing sleep and sleep problems (from CDC-SI), sleep disturbance and sleep-related impairment (from PROMIS Sleep Short Forms), and a variety of measures from DSQ items 19–24 (unrefreshed, need to nap, problems falling asleep, problems staying asleep, awaking too early, and sleep all day/awake all night). The mean scores by site for nearly all measures show no statistically significant difference. The two exceptions are PROMIS sleep-related impairment and DSQ unrefreshed. The T-scores for PROMIS sleep-related impairment means by site ranged from 60.5 (SD = 7.1) to 64.3 (SD = 7.6), a difference of 3.8 points that would be considered clinically meaningful [13]. However, the SD of the measures is nearly twice the difference in the site means. Both the CDC SI and DSQ measure unrefreshing sleep using a similar Likert scale. Although the CDC-SI measure of unrefreshing sleep was not significantly different across sites, it showed the same trends by site as DSQ unrefreshing sleep. 

#### 3.3.5. Measures of Neurocognitive/Autonomic Symptoms

Table 8 shows the mean scores by site and overall for the four symptoms measured in the CDC-SI (memory, concentration, sensitive to light, and short of breath) and the twenty symptoms measured in the DSQ (items 32–51). The mean symptom scores by site did not differ statistically for nearly all these measures. The two exceptions are DSQ items 34 (noise sensitive, range of mean scores of 4.5 to 8.4) and 48 (unsteady on feet, range of mean scores of 2.7 to 5.2). The sites all showed similarities in the symptoms with the highest and lowest mean scores. The four DSQ symptoms with the highest mean scores were the same for all sites and overall, i.e., concentration, memory, only focus on one thing, and word-finding difficulty. Memory and concentration were the CDC-SI symptoms with the highest mean scores in all sites and overall. The three DSQ scores with the lowest mean scores were the same for all sites and overall, i.e., irregular heartbeats, loss depth perception, and nausea. In addition, for all but one site, muscle twitch was among the four DSQ symptoms with lowest mean scores.

#### 3.3.6. Measures of Pain

Table 9 shows the mean scores by site and overall for the pain-related measures, those in the Brief Pain Inventory (BPI), PROMIS Pain Interference and Pain Behavior, three CDC-SI symptoms, and DSQ items #25–31. Once again, there were only a few items with statistically significant differences in means across sites (severity of pain in BPI, joint pain in CDC-SI, muscle pain or ache and pain/stiffness without swelling in >1 joint in DSQ). The PROMIS T-scores for pain interference were slightly higher than pain behavior in each site. In both the CDC-SI and DSQ, muscle symptoms have higher mean scores in each site than joint symptoms. 

#### 3.3.7. Measures of Other Symptoms

Table 10 shows the mean scores by site and overall for symptoms classified as immunologic/inflammation, gastrointestinal, and emotional or behavioral. Immunologic/inflammation symptoms include five measures from the CDC-SI (sore throat, sinus problems, tender lymph nodes, fever, chills) and five from the DSQ (items 62–66). The mean scores by site show significant differences only for tender lymph nodes in the CDC SI (mean scores range from 2.4 to 4.9) and fever in the DSQ (mean scores range from 0.6 to 1.7). The pattern of symptom severity (as measured by mean scores) was nearly identical among the sites and overall. The top two CDC SI immunologic/inflammation symptoms were sinus problems and tender lymph nodes (all but one site had higher mean scores for sinus problems), whereas fever was the symptom with the lowest mean scores. The top two DSQ symptoms in the immunologic/inflammatory group were sickened by smells, food, medications, chemicals, and flu-like symptoms for all sites and overall, whereas fever was the symptom with the lowest mean scores. Measures of gastrointestinal symptoms did not differ between sites. The mean score for stomach pain was higher than for diarrhea for all sites and overall. 

None of the measures of depression, anxiety, or mentally unhealthy days differed between clinics and overall. Overall, the mean scores for Zung SDS (45.0, SD = 9.0), GAD-7 Anxiety (5.1, SD = 5.3), CDC HRQoL mentally unhealthy days (9.2, SD = 10.5), and PHQ-8 (10.0, SD = 5.1) indicate that depression and anxiety are clinically significant issues for some study participants.

#### 3.3.8. Proportion Meeting Case Definitions

**Figure 3:**
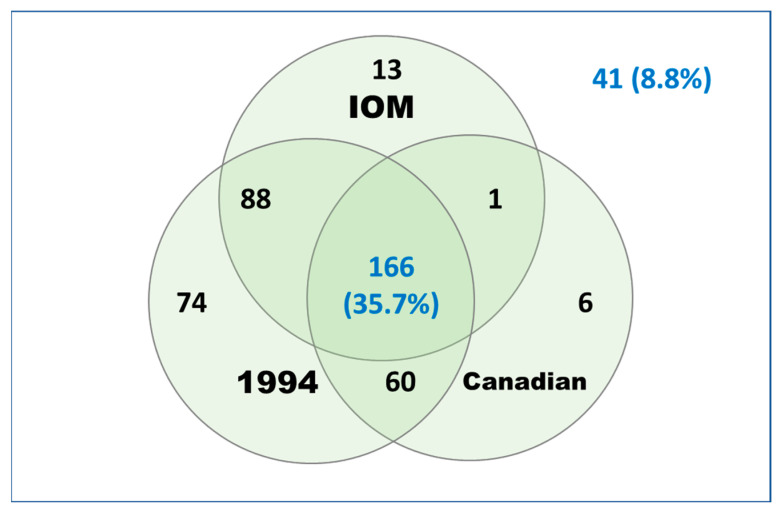
Agreement in classification by case definition algorithm. Venn diagram showing the overlap in classification by case definition algorithm. The number that did not fulfil any of the algorithms is shown in the background. Note: Data exclude 16 participants with insufficient information to determine all classifications.

Table 11 shows the proportion of patients meeting each of the three case definition algorithms by site and overall. For each site, the 1994 research algorithm was met by the highest proportion of patients (83.4% overall, range of 77.0–90.0), and the 2003 Canadian algorithm was met by the lowest proportion of patients (50.1% overall, range of 45.9–57.4). Figure 3 illustrates the agreement between case definition algorithms for the 449 patients with data for all three calculations. Of note, 352 (70.2%) met two or more algorithms, and 41 patients (9.1%) did not meet any of the algorithms. Two-way concordance between algorithms was similar: 1994 and Canadian, 0.624; 1994 and IOM, 0.670; and Canadian and IOM, 0.628. 

#### 3.3.9. Histograms of CDC SI Scores

**Figure 4:**
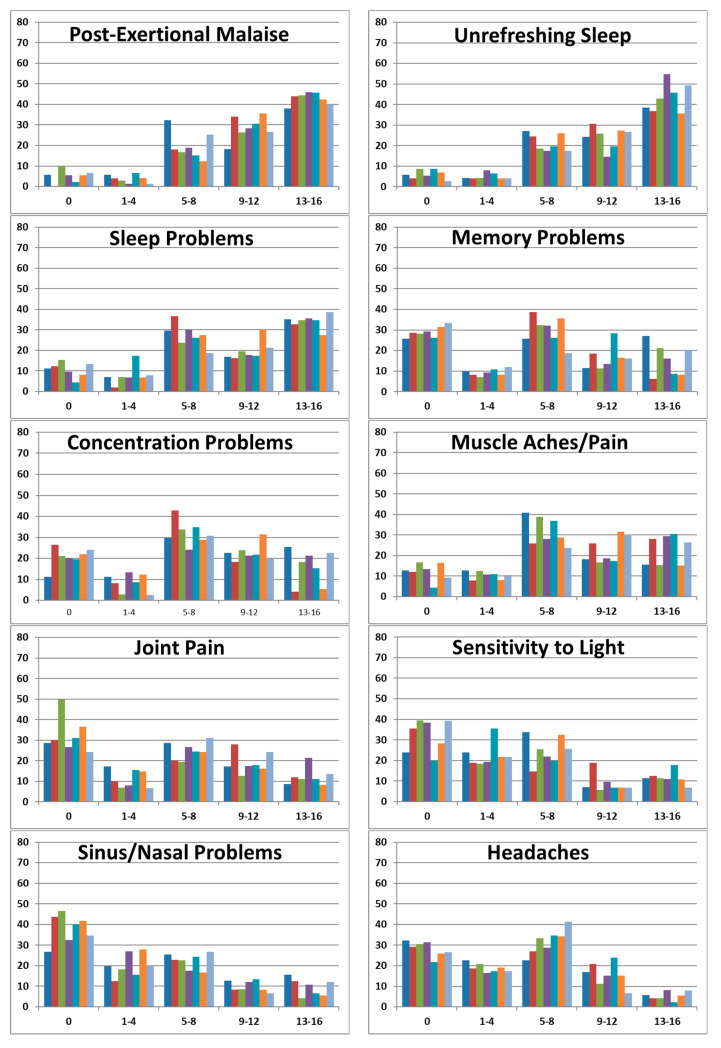

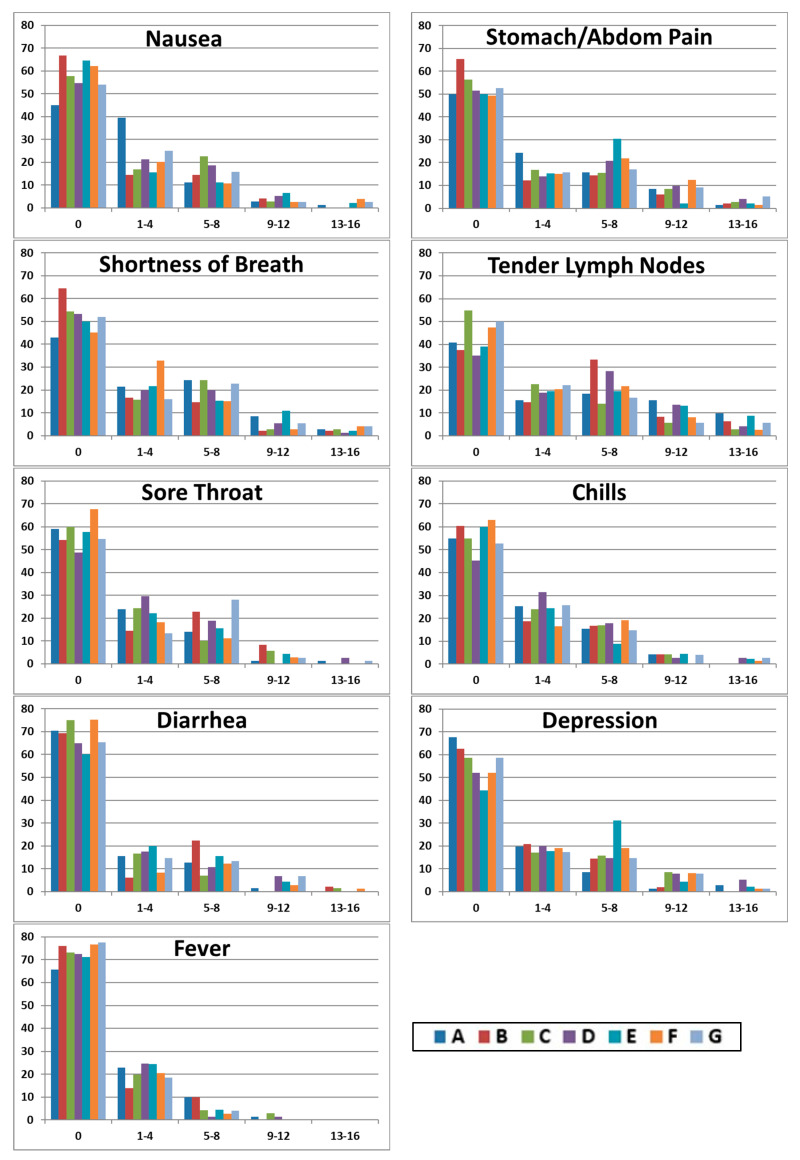
Histograms of CDC Inventory Scores by clinic. Frequency of CDC Symptom Inventory scores (frequency X severity) is shown by score groups 0 (not present), 1–4, 5–8, 9–12, and 13–16 (highest scores) for each clinic (A–G, shown by colors noted at the bottom of the figure).

As noted above, the standard deviations of the measures of illness indicate the heterogeneity of these measures. Histograms of the CDC Symptom Inventory scores (Figure 4) illustrate how score distributions differ by symptom, with each clinic’s scores following the same pattern. Symptoms included in most case definitions (post-exertional malaise and sleep) have greater frequency in the higher symptom score groups. By comparison, the score distributions of memory and concentration, muscle aches/pain, joint pain, sensitivity to light, and sinus/nasal problems are more evenly spread over the score groups. Other symptoms, such as fever, chills, and depression, are heavily represented in the lower score groups. The DSQ scores follow the same pattern.

## 4. Discussion

We initiated this study to characterize patients with ME/CFS based on the clinical opinion of clinicians with recognized expertise in diagnosing and caring for these patients. The goal of this analysis was to determine if differences in clinical practice resulted in different subgroups of patients. We used standardized instruments to measure the major domains of illness experienced by patients with ME/CFS and evaluated these characteristics for patients seen at each clinic and overall. The specialty clinics differed in size (solo practice, small group, academic, hospital-based), location (metropolitan, urban, and rural), and practice characteristics (time spent with patients, charges for initial visit, follow-up visits). The board certifications of the physicians included internal medicine, environmental medicine, infectious diseases, immunology, hematology, neurology, and pediatrics. Certifications could influence referral patterns and approaches to laboratory testing, diagnosis, and management. There were statistical differences between sites in the general characteristics of their patients. Socio-demographic variables (Table 2) showed statistical differences by clinic site, but the distributions of the variables overlapped (such as age at enrollment, Figure 1) and were in the same direction (predominately insured white women with a high level of education). Similarly, there were statistical differences by site in the age at diagnosis, mode of onset, duration of fatigue, and BMI. Again, distributions were overlapping (such as age at diagnosis, Figure 2) and in the same direction (majority of patients had sudden onset and were overweight). Interestingly, the largest variation was in the number of medications prescribed for patients, with a mean ranging from 4.1 to 8.2 (*p* = 0.001). 

In the face of these practice differences, it is striking that we found few statistically significant and no clinically meaningful differences between clinics in their patients’ standardized measures of ME/CFS symptoms and function (Table 4, Table 5, Table 6, Table 7, Table 8, Table 9 and Table 10). This suggests that expert clinicians are recognizing the same clinical entity, albeit one that is far from homogeneous. Importantly, patients in each clinic sample and overall showed a wide distribution in all scores and measures. The data tables are provided as a reference of these illness measures in clinically well-characterized patients.

Heterogeneity in ME/CFS illness characteristics has been recognized, and researchers have been advised to use more restrictive or overlapping case definitions to improve case ascertainment [14]. However, symptom-based case definitions have limitations, including a lack of guidance in how each criterion of the definition should be met. Differences in how the same case definition is applied or operationalized can impact case ascertainment [5]. The use of common data elements for ME/CFS [8] will improve reproducibility, but work remains in establishing the optimal thresholds for each measure. However, current ME/CFS case definitions do not eliminate heterogeneity. For example, the Chronic Fatigue Initiative [15] required that the 203 enrolled patients with ME/CFS met the 1994 research case definition [9], the 2003 Canadian definition [10], or both and reported SF-36 scores with standard deviations similar to those observed in our study [15]. 

The medical complexity of ME/CFS and the lack of objective diagnostic tests presents challenges for case ascertainment in research and clinical care. Heterogeneity is masked when studies report means or medians. Presenting research data in a format that shows heterogeneity, such as scatter plots or histograms, will help clarify the challenge. Variations in patient demographics, co-morbid conditions, medications, and duration of illness can all contribute to heterogeneity [16]. 

ME/CFS shares many characteristics with post-acute infection syndromes (PAISs) and is often considered to have an infectious trigger, although other triggers are not ruled out. The COVID-19 pandemic, resulting in a chronic PAIS termed Long COVID, has increased clinical and research studies seeking to understand the mechanisms and options for treatments. Despite a single defined trigger (SARS CoV-2), patients with Long COVID have striking heterogeneity in their illness profiles. An early report from the Patient-Led Research Collaborative [17] collected data on 203 symptoms in 10 organ systems. Illness heterogeneity may be an inherent feature of these syndromes. Relying on case–control study designs without the subgrouping or stratification of ME/CFS illness characteristics may limit the reproducibility of research findings and could obscure underlying mechanisms. Study designs that compare similarities and differences in ME/CFS, Long COVID, and other PAIS and begin to link biomarkers to symptom measures and subgroups may be needed.

The standardized scores demonstrate that, despite expert clinical care, the burden of illness experienced by patients with ME/CFS is significant. The overall mean for the number of physically unhealthy (23.2, SD = 9.5, Table 4) and mentally unhealthy days (9.2, SD = 10.5, Table 10) in the past 30 days are much higher than the 2017 U.S. average (4.0 days for each score; https://www.shadac.org/news/adult-unhealthy-days-new-measure-state-health-compare, accessed on 21 February 2024). The mean PHQ-8 score (10.0, SD = 5.1) and Zung score (45.0, SD = 5.3) indicate that many patients experience clinically significant depression (Table 10; PHQ-8 score ≥ 10 represents clinically significant depression; Zung score 40–47 indicates mild depression). 

While post-exertional malaise (PEM) is considered to be characteristic of ME/CFS and is a required symptom in the 2015 IOM clinical case definition [6], methods to identify PEM are not standardized. We found that the five DSQ items recently recommended as a first step in identifying PEM [18] correlated well with results based on a single question in the CDC SI (92.4%, 426/461). Cotler et al. found that supplementary questions on the timing and duration of PEM were necessary to distinguish between ME/CFS and other illnesses [18], and this could not be evaluated in our study. Given the burden of symptom reporting, we suggest that further evaluation of the single PEM question in the CDC SI is warranted. 

This study’s strength lies in the clinical expertise and combined data from seven ME/CFS specialty clinics. However, this strength is also a limitation as those seen in other clinics, by primary care physicians, or not having access to any care are not represented. This could bias the study sample and may limit the generalizability of the findings. Patients with illness severity that prevents travel to clinics were not included, and this should be kept in mind when interpreting the results. The study population is largely white, highly educated, and insured. Increasing patient diversity would be unlikely to decrease the heterogeneity of illness characteristics; thus, the major observation that ME/CFS is a heterogenous illness and that the heterogeneity is not explained by different clinical practices remains. Study designs that incorporate illness heterogeneity to shed light on underlying pathogenesis are needed.

## Figures and Tables

**Table 1:** Data sources for measures of ME/CFS illness domains [7].

Data Sources for Measures of Illness Domains
Functional impairment Medical Outcomes Study 36-Item Short Form Survey (SF-36) v2 Hours Vertical Report of Exercise CDC Health-related Quality of Life (HRQoL) (physically unhealthy days)
Post-exertional malaise Illness Abstraction Form (IAF) (medical records abstraction) CDC Symptom Inventory (PEM) DePaul Symptom Questionnaire (items 14–18)
Fatigue 20-item Multi-dimensional Fatigue Inventory (MFI-20) PROMIS Fatigue Short Form 7a (PROMIS F-SF 7a)
Sleep problems CDC Symptom Inventory (unrefreshing sleep, sleep problems) PROMIS Sleep Disturbance 8-Item Short Form PROMIS Sleep-Related Impairment Short Form v1.0 DePaul Symptom Questionnaire (items 19–24)
Neurocognitive/Autonomic Symptoms CDC Symptom Inventory (memory, concentration, sensitive to light, short of breath) DePaul Symptom Questionnaire (items 32–51)
Pain Brief Pain Inventory (long form) PROMIS Pain Interference Short Form 8a (PROMIS PI-SF 8a) PROMIS Pain Behavior Short Form 8a (PROMIS PB-SF 8a) CDC Symptom Inventory (headaches, joint pain, muscle aches) DePaul Symptom Questionnaire (items 25–31)
Other symptoms *Immunologic/inflammation* CDC Symptom Inventory (sore throat, sinus problems, tender lymph nodes, fever, chills) DePaul Symptom Questionnaire (items 62–66) *Gastrointestinal* CDC Symptom Inventory (stomach/abdominal pain, diarrhea) *Emotional or behavioral* CDC Symptom Inventory (Depression) Zung Self-Rating Depression Scale (SDS) 7-item Generalized Anxiety Disorder (GAD-7) CDC Health-Related Quality of Life (HRQoL) (mentally unhealthy days) 8-item Patient Health Questionnaire (PHQ-8)

**Table 2:** Socio-demographic characteristics of study population by site (A through G) and overall.

	A (n = 71, 15.3%)	B (n = 50, 10.8%)	C (n = 72, 15.5%)	D (n = 75, 16.1%)	E (n = 46, 9.9%)	F (n = 74, 15.9%)	G (n = 77, 16.7%)	Overall (n = 465)
Age at enrollment, yrs, mean (SEM) ***	51.3 (13.3)	52.5 (8.5)	43.3 (14.1)	47.5 (10.5)	51.5 (9.7)	43.6 (14.8)	48.5 (11.5)	47.9 (12.6)
Sex *								
Male	27 (38%)	10 (20%)	25 (34.7%)	14 (18.7%)	7 (15.2%)	21 (28.4%)	16 (20.8%)	120 (25.8%)
Female	44 (62%)	40 (80%)	47 (65.3%)	61 (81.3%)	39 (84.8%)	53 (71.6%)	61 (79.2%)	345 (74.2%)
Female–male ratio	1.6	4.0	1.9	4.4	5.6	2.5	3.8	2.9
Race ***								
White	70 (98.6%)	47 (94%)	65 (90.3%)	75 (100%)	45 (97.8%)	72 (97.3%)	65 (84.4%)	439 (94.4%)
Black/African American	0	1 (2%)	0	0	1 (2.2%)	0	4 (5.2%)	6 (1.3%)
All others	1 (1.4%)	2 (4%)	7 (9.7%)	0	0	2 (2.7%)	8 (10.4%)	20 (4.3%)
Marital status **								
Married/committed relationship	41 (58.6%)	30 (60%)	37 (53.6%)	42 (57.5%)	33 (73.3%)	42 (57.5%)	31 (40.8%)	256 (56.1%)
Previously married	13 (18.6%)	14 (28%)	9 (13%)	12 (16.4%)	4 (8.9%)	6 (8.2%)	17 (22.4%)	75 (16.5%)
Never married	16 (22.9%)	6 (12%)	23 (33.3%)	19 (26%)	8 (17.8%)	25 (34.2%)	28 (36.8%)	125 (27.4%)
Employment								
Full-time	9 (12.9%)	8 (16.3%)	10 (14.3%)	13 (17.3%)	3 (6.5%)	6 (8.2%)	17 (22.1%)	66 (14.4%)
Part-time	8 (11.4%)	5 (10.2%)	15 (21.4%)	8 (10.7%)	2 (4.3%)	6 (8.2%)	7 (9.1%)	51 (11.1%)
Not employed	53 (75.7%)	36 (73.5%)	45 (64.3%)	54 (72%)	41 (89.1%)	61 (83.6%)	53 (68.8%)	343 (74.6%)
Insurance **								
Yes	64 (95.5%)	50 (100%)	62 (89.9%)	75 (100%)	40 (87%)	68 (91.9%)	71 (94.7%)	430 (94.3%)
No	3 (4.5%)	0	7 (10.1%)	0	6 (13%)	6 (8.1%)	4 (5.3%)	26 (5.7%)
Education ***								
Less than high school	0	0	0	1 (1.5%)	0	3 (4.1%)	0	4 (0.9%)
High school graduate	18 (26.5%)	9 (18%)	10 (14.1%)	17 (25%)	6 (13.3%)	27 (37%)	14 (18.2%)	101 (22/4%)
College graduate	22 (32.4%)	26 (52%)	28 (39.4%)	23 (33.8%)	19 (42.2%)	32 (43.8%)	28 (36.4%)	178 (39.4%)
Post-college	28 (41.2%)	15 (30%)	33 (46.5%)	27 (39.7%)	20 (44.4%)	11 (15.1%)	35 (45.5%)	169 (37.4%)

All values are No. (Column %) unless otherwise indicated. *** *p* < 0.001; ** *p* < 0.01; * *p* < 0.05.

**Table 3:** General clinical characteristics of patients by site (A through G) and overall.

	A (n = 71, 15.3%)	B (n = 50, 10.8%)	C (n = 72, 15.5%)	D (n = 75, 16.1%)	E (n = 46, 9.9%)	F (n = 74, 15.9%)	G (n = 77, 16.7%)	Overall (n = 465)
Age at diagnosis (yrs) **	37.3 (11)	38.9 (9.9)	37.6 (14)	40.3 (11.4)	39.0 (11)	33.5 (12.2)	40.9 (11.9)	38.2 (12.6)
Mode of onset **								
Sudden	46 (75.4%)	22 (52.4%)	48 (72.7%)	41 (63.1%)	27 (67.5%)	47 (74.6%)	33 (49.3%)	264 (65.4%)
Gradual	15 (24.6%)	20 (47.6%)	18 (27.3%)	24 (36.9%)	13 (32.5%)	16 (25.4%)	34 (50.7%)	140 (34.6%)
Duration of fatigue (yrs) ***	17.9 (9.9)	16.5 (8.3)	9.4 (8.0)	10.9 (9.6)	17.8 (10.8)	13.5 (8.9)	14.7 (10.6)	14.1 (9.9)
BMI (kg/m^2^) **	26.2 (4.5)	28.8 (7.6)	24.3 (4.9)	26.6 (6.1)	29.2 (7)	25.5 (5.7)	25.9 (7.3)	26.6 (6.3)
Number of medications ***	5.6 (4.2)	6.5 (4.7)	4.1 (3.4)	6.4 (4.4)	8.2 (4.8)	6.4 (4.5)	5.0 (4.1)	5.9 (4.4)

All values are mean (standard deviation) unless otherwise indicated. *** *p* < 0.001; ** *p* < 0.01; * *p* < 0.05.

**Table 4:** Measures of functional impairment by site (A through G) and overall.

	A (n = 71, 15.3%)	B (n = 50, 10.8%)	C (n = 72, 15.5%)	D (n = 75, 16.1%)	E (n = 46, 9.9%)	F (n = 74, 15.9%)	G (n = 77, 16.7%)	Overall (n = 465)
**SF-36 (0–100)**								
Physical Functioning	38.9 (27.8)	41 (23.1)	38.8 (24.8)	36.8 (22.5)	35.2 (25.2)	38.7 (20.1)	39.9 (19.7)	38.5 (23.3)
Role Physical	7.4 (22.9)	4.5 (12.0)	4.0 (18.0)	6.3 (20.2)	7.2 (22.4)	3.8 (14.8)	4.0 (15.4)	5.2 (18.3)
Bodily Pain	39.2 (25.3)	34.7 (24.1)	41.6 (24.2)	37.2 (26.0)	36.3 (24.1)	37.3 (23.4)	33.9 (20.8)	37.3 (24.0)
General Health	25.1 (19.2)	28.5 (18.5)	23.7 (13.6)	24.8 (16.6)	28.4 (16.7)	24.5 (13.4)	27.7 (17.1)	25.9 (16.5)
Vitality/energy/fatigue	20.7 (21.0)	19.1 (14.9)	15.9 (18.5)	14.3 (16.3)	17.4 (21)	18.1 (14.4)	15.5 (12.0)	17.1 (17.0)
Social Functioning	26.6 (26.4)	31.9 (20.1)	21.7 (24.3)	23.6 (23.5)	27.2 (28.8)	26.4 (21.8)	25.2 (18.5)	25.7 (23.4)
Role Emotion	82.8 (36.6)	74.7 (41.3)	70.4 (43.5)	69.8 (42.4)	62.3 (45.9)	62.6 (44.8)	73.6 (40.6)	71.1 (42.4)
Mental Health	69.6 (22.3)	67.3 (20.3)	61.4 (20.4)	65 (21.8)	66.1 (24.5)	65.5 (20.5)	66.1 (20.1)	65.7 (21.3)
**Hours of Vertical Activity (0–15)**	7.8 (4.2)	8.0 (3.7)	7.3 (4.7)	7.9 (4.5)	6.3 (4.1)	7.5 (3.7)	7.5 (4.1)	7.5 (4.2)
**Exercise (Yes/No)**								
Strenuous exercise	7 (10.9%)	4 (10.0%)	8 (11.6%)	5 (6.7%)	2 (4.6%)	5 (7.7%)	3 (4.1%)	34 (7.8%)
Moderate exercise	19 (28.4%)	16 (36.4%)	21 (30.4%)	17 (23.3%)	11 (25.0%)	22 (32.8%)	23 (31.1%)	129 (29.5%)
Mild exercise	40 (66.7%)	37 (84.1%)	49 (71.0%)	47 (65.3%)	32 (71.1%)	53 (75.7%)	60 (83.3%)	318 (73.6%)
**CDC Health-related QoL (0–30 days)**								
Physically unhealthy days	22.4 (10.0)	20.6 (9.7)	25.1 (9.1)	24.8 (9.4)	21.8 (9.2)	20.5 (9.5)	25.0 (9.1)	23.2 (9.5)

All values are mean (standard deviation) unless otherwise indicated. No comparisons were statistically significant at *** *p* < 0.001, ** *p* < 0.01 or * *p* < 0.05.

**Table 5:** Measures of post-exertional malaise (PEM) by site (A through G) and overall.

	A (n = 71, 15.3%)	B (n = 50, 10.8%)	C (n = 72, 15.5%)	D (n = 75, 16.1%)	E (n = 46, 9.9%)	F (n = 74, 15.9%)	G (n = 77, 16.7%)	Overall (n = 465)
**Illness Abstraction Form ^1^**								
PEM present (%)	60 (84.5%)	44 (88.0%)	63 (87.5%)	68 (90.7%)	40 (87.0%)	62 (83.8%)	67 (87.0%)	421 (87.5%)
**CDC SI (score range 0–16) ^1^**								
PEM score, 0–16	10.8 (5.1)	12.6 (3.3)	11.5 (5.3)	12.1 (4.7)	12.1 (4.7)	11.8 (4.8)	11.4 (4.9)	11.7 (4.8)
PEM present (%) ^2^	67 (94.4%)	50 (100%)	65 (90.3%)	70 (94.6%)	45 (97.8%)	69 (94.5%)	70 (93.3%)	446 (94.7%)
**DePaul Symptom Questionnaire (DSQ) (score range 0–16) ^1^**								
14—Dead/heavy feeling after starting exercise	8.3 (5.3)	8.2 (5.8)	8.9 (5.3)	8.8 (5.9)	8.9 (6)	8.4 (5.3)	7.6 (5.6)	8.4 (5.5)
15—Sore/fatigue next day after usual activity	7.4 (4.9)	7.9 (5.6)	8.1 (5.2)	9.2 (5.7)	8.5 (5.5)	7.5 (4.5)	9.1 (5.1)	8.3 (5.2)
16—Mentally tired after slightest effort	6.4 (5.1)	5.8 (5.2)	7.3 (4.9)	7.1 (5.6)	6.8 (5.3)	5.2 (4.4)	7 (5.1)	6.5 (5.1)
17—Tired after minimal exercise	7.9 (5.7)	8.3 (5.1)	9.5 (5.3)	10.1 (5.6)	9.5 (5.6)	8.9 (4.9)	9.4 (5.1)	9.1 (5.4)
18—Physically drained or sick after mild activity	7.2 (5.6)	7.4 (5.6)	8.4 (5.4)	8.5 (5.4)	8.3 (5.5)	7.1 (4.4)	8.4 (5.4)	7.9 (5.3)
PEM present (%) ^3^	64 (90.1%)	48 (96.0%)	69 (95.8%)	69 (92.0%)	41 (89.1%)	71 (96.0%)	75 (97.4%)	437 (94.0%)

All values are mean (standard deviation) unless otherwise indicated. No comparisons were statistically significant at *** *p* < 0.001, ** *p* < 0.01 or * *p* < 0.05. ^1^ Insufficient information was available to determine PEM status using all three approaches for four subjects. ^2^ PEM present for CDC SI defined as experienced ≥ 6 months and present during the past month. ^3^ PEM present for DSQ if frequency ≥ 2 (“About Half the Time”) and severity ≥ 2 (“Moderate”) for at least one of DSQ items #14–#18.

**Table 6:** Measures of fatigue by site (A through G) and overall.

	A (n = 71, 15.3%)	B (n = 50, 10.8%)	C (n = 72, 15.5%)	D (n = 75, 16.1%)	E (n = 46, 9.9%)	F (n = 74, 15.9%)	G (n = 77, 16.7%)	Overall (n = 465)
**MFI-20 (score range 4–20)**								
General Fatigue	17.7 (3.0)	18.1 (2.3)	18.4 (2.0)	18.6 (2.1)	18.1 (2.8)	18.2 (2.2)	18.5 (1.5)	18.2 (2.3)
Physical Fatigue *	16.9 (3.5)	16.5 (3.0)	17.9 (2.7)	17.7 (2.7)	17 (3.3)	17.9 (2.2)	17.7 (2.3)	17.4 (2.8)
Mental Fatigue	14.3 (4.1)	14.1 (4.2)	15.2 (4.2)	14.9 (4.1)	14.7 (3.8)	14.7 (3.4)	15.2 (4.0)	14.8 (4.0)
Reduced Activity *	15.1 (3.8)	15.3 (3.8)	17.2 (2.9)	16.2 (4.0)	16.4 (3.9)	16.2 (3.4)	16.2 (3.3)	16.1 (3.6)
Reduced Motivation	11.4 (3.7)	11.5 (4.8)	11.6 (4.7)	12.1 (4.1)	13.7 (4.2)	11.9 (3.7)	12.3 (4.3)	12.0 (4.2)
**PROMIS Fatigue**								
T-Score (range 29.4–83.2)	67.0 (8.4)	67.4 (5.9)	68.7 (7.4)	68.5 (7.4)	69.7 (8.1)	67.9 (5.3)	68.9 (6.4)	68.3 (7.0)

All values are mean (standard deviation). *** *p* < 0.001; ** *p* < 0.01; * *p* < 0.05.

**Table 7:** Measures of sleep by site (A though G) and overall.

	A (n = 71, 15.3%)	B (n = 50, 10.8%)	C (n = 72, 15.5%)	D (n = 75, 16.1%)	E (n = 46, 9.9%)	F (n = 74, 15.9%)	G (n = 77, 16.7%)	Overall (n = 465)
**CDC-SI (score range 0–16)**								
Unrefreshing sleep	11.1 (5.0)	11.3 (4.7)	11.3 (5.3)	11.8 (5.5)	11.1 (5.6)	10.8 (5.0)	12.4 (4.5)	11.4 (5.1)
Sleeping problems	9.8 (5.6)	9.8 (5.5)	9.6 (6.0)	9.8 (5.6)	9.8 (5.7)	10.1 (5.1)	10.1 (6)	9.9 (5.6)
**PROMIS Sleep**								
Sleep disturbance T-score (range 28.4–83.2)	59.5 (8.2)	59.7 (8.0)	59.3 (8.4)	59.3 (8.3)	59.5 (8.8)	58.5 (7.7)	60.4 (7.6)	59.4 (8.1)
Sleep-related impairment T-score (range 30.0–80.0) *	61.5 (8.4)	64.2 (6.7)	62 (8.4)	61.6 (8.6)	61.9 (8.6)	60.5 (7.1)	64.3 (7.6)	62.2 (8.0)
**DePaul Symptom Questionnaire (score range 0–16)**								
19—Unrefreshed *	9.6 (5.1)	10.4 (5.5)	10.8 (4.9)	10.9 (5.3)	9.9 (5.9)	9.4 (4.7)	11.8 (4.3)	10.4 (5.1)
20—Need to nap	5.7 (5.5)	7.1 (5.5)	6.3 (5.8)	7 (5.8)	7.6 (5.6)	5.2 (5)	7.1 (5.8)	6.5 (5.6)
21—Problems falling asleep	7.4 (5.7)	6 (5.2)	6.6 (5.5)	6.5 (6)	7.2 (5.6)	7.2 (5.4)	8 (6)	7.0 (5.7)
22—Problems staying asleep	6.6 (5.2)	6.5 (5.7)	6.1 (5.6)	6.8 (6.3)	6.9 (6.2)	5.9 (5.3)	6.9 (6)	6.5 (5.7)
23—Awaking too early	6.2 (5.7)	6 (5.4)	4.4 (5.4)	5.7 (6.1)	5.8 (6.2)	3.9 (4.5)	4.9 (5.3)	5.2 (5.5)
24—Sleep all day/awake all night	1.5 (3.5)	1.3 (3.4)	1.9 (3.7)	1.4 (2.9)	1.2 (2.2)	2.0 (3.1)	1.4 (3.5)	1.5 (3.3)

All values are mean (standard deviation). *** *p* < 0.001; ** *p* < 0.01; * *p* < 0.05.

**Table 8:** Measures of neurocognitive/autonomic symptoms by site (A through G) and overall.

	A (n = 71, 15.3%)	B (n = 50, 10.8%)	C (n = 72, 15.5%)	D (n = 75, 16.1%)	E (n = 46, 9.9%)	F (n = 74, 15.9%)	G (n = 77, 16.7%)	Overall (n = 465)
**CDC-SI (score range 0–16)**								
Memory	7.7 (6.3)	6.1 (5.0)	7.2 (6.0)	6.5 (5.8)	6.9 (5.4)	6.0 (5.3)	6.7 (6.3)	6.7 (5.8)
Concentration	8.9 (5.6)	6.0 (4.7)	8.3 (5.5)	7.7 (6.0)	7.7 (5.4)	6.9 (5.2)	8.3 (5.9)	7.8 (5.6)
Sensitive to light	5.5 (5.2)	5.6 (5.8)	4.5 (5.4)	4.7 (5.4)	5.7 (5.8)	5.2 (5.2)	4.0 (4.8)	5.0 (5.3)
Short of breath	3.6 (4.5)	1.9 (3.6)	2.6 (3.9)	2.5 (3.8)	3.0 (4.4)	2.7 (4.0)	3.1 (4.4)	2.8 (4.1)
**DePaul Symptom Questionnaire (score range 0–16)**								
32—Muscle twitch	2.7 (3.6)	3.1 (4.3)	2.3 (3.6)	2.3 (3.2)	3.2 (3.5)	1.8 (2.9)	2.8 (3.5)	2.5 (3.5)
33—Muscle weak	6.1 (5)	5.6 (5.2)	6.0 (5.1)	6.1 (5.6)	7.8 (5.4)	5.6 (4.5)	6.4 (4.6)	6.2 (5.0)
34—Noise sensitive **	6.1 (5.1)	6.5 (5.4)	5.6 (4.9)	4.5 (5)	8.4 (5.5)	5.8 (4.9)	5.5 (4.8)	5.9 (5.1)
35—Bright light sensitive	5.9 (5.1)	6.2 (5.8)	4.9 (4.8)	5.5 (5.3)	7.6 (5.9)	5 (4.9)	4.9 (4.6)	5.6 (5.2)
36—Memory	7.2 (5.5)	7.0 (4.5)	7.5 (5.5)	7.0 (5.6)	7.6 (4.6)	5.8 (4.1)	7.2 (5.2)	7.0 (5.1)
37—Concentration	8.2 (5.4)	6.3 (5.3)	8.1 (5.5)	8.5 (5.7)	7.9 (5.4)	6.5 (4.4)	8.2 (5.4)	7.7 (5.3)
38—Word-finding difficulty	6.8 (5.4)	6.2 (5.1)	6.8 (5)	6.5 (5.9)	7.2 (5.4)	6.1 (4.1)	7.2 (5.2)	6.7 (5.1)
39—Difficulty understanding	5.0 (5)	3.5 (4)	4.5 (4.7)	3.8 (4.9)	5.2 (4.9)	3.4 (3)	3.9 (4.3)	4.2 (4.4)
40—Only focus on one thing	7.1 (5.1)	6.2 (5)	7.7 (5.7)	7.4 (5.3)	8.1 (5.6)	5.6 (4.8)	6.9 (5)	7.0 (5.2)
41—Unable to focus vision/attention	4.7 (5.3)	3.6 (4.4)	5.7 (5.3)	4.4 (5)	5.4 (5.3)	3.5 (3.8)	4.9 (4.8)	4.6 (4.9)
42—Loss depth perception	2.6 (4.3)	2.4 (4.4)	2.8 (4.6)	1.9 (3.8)	3.3 (4.5)	1.3 (2.8)	1.5 (3.1)	2.2 (3.9)
43—Slow thought	6.5 (5.6)	4.7 (4.5)	6.7 (5.4)	5.5 (5.5)	6.1 (5)	4.7 (4.3)	5.7 (5)	5.7 (5.1)
44—Forgetful	6.6 (5.6)	5.8 (4.7)	7.3 (5.6)	6.5 (5.5)	7.3 (5.4)	5.1 (4.3)	6 (5.3)	6.3 (5.3)
45—Bladder problems	3.4 (4.9)	3.6 (5.4)	2.9 (4.6)	3.9 (5.1)	4 (4.9)	2.1 (3.2)	2.8 (4)	3.2 (4.6)
46—Irritable bowel problems	3.8 (4.7)	5.0 (6)	3.4 (4.9)	4.8 (5.8)	4.8 (5.5)	3.9 (4.6)	4.3 (4.7)	4.2 (5.1)
47—Nausea	1.9 (2.7)	1.7 (3.2)	2.4 (3.3)	2.4 (3.5)	2.3 (3.2)	2.5 (4.2)	1.9 (2.9)	2.2 (3.3)
48—Unsteady on feet *	2.7 (3.4)	2.9 (4.1)	3.6 (4.1)	3.1 (3.7)	5.2 (5.3)	2.9 (3.2)	3.3 (3.8)	3.3 (3.9)
49—Shortness of breath	3.5 (4.6)	2.1 (3.9)	3.2 (4)	2.7 (3.9)	3.4 (4.3)	2.6 (3.5)	3.2 (4.2)	3.0 (4.1)
50—Dizziness or fainting	2.8 (3.4)	2 (3.3)	3.3 (3.6)	3.2 (3.6)	2.8 (3.7)	2.9 (3.2)	2.6 (2.9)	2.8 (3.4)
51—Irregular heart beats	2.0 (3)	1.3 (2.1)	1.9 (3)	2.1 (3.3)	1.6 (2.5)	1.5 (2.1)	1.5 (2.9)	1.7 (2.8)

All values are mean (standard deviation). *** *p* < 0.001; ** *p* < 0.01; * *p* < 0.05.

**Table 9:** Measures of pain by site (A through G) and overall.

	A (n = 71, 15.3%)	B (n = 50, 10.8%)	C (n = 72, 15.5%)	D (n = 75, 16.1%)	E (n = 46, 9.9%)	F (n = 74, 15.9%)	G (n = 77, 16.7%)	Overall (n = 465)
**Brief Pain Inventory (score range 0–10)**								
Interference of pain	4.6 (0.4)	5.3 (0.4)	4.1 (0.4)	5.1 (0.3)	5.1 (0.4)	4.9 (0.3)	5 (0.3)	4.8 (0.1)
Severity of pain **	3.8 (0.3)	4.7 (0.4)	3.4 (0.3)	4.8 (0.3)	4.5 (0.3)	4.1 (0.3)	4.9 (0.3)	4.3 (0.1)
**PROMIS**								
Pain Interference T-Score (range 41.0–78.3)	61.1 (1.1)	63.9 (1.3)	59.6 (1.2)	62.2 (1.2)	64.1 (1.4)	62.5 (1)	62.8 (1.1)	62.2 (0.4)
Pain Behavior T-Score (range 36.7–75.9)	56.9 (0.9)	58.6 (1)	55.5 (1)	56.3 (1)	58.8 (1.1)	58 (0.8)	58 (0.8)	57.3 (0.3)
**CDC-SI (score range 0–16)**								
Headaches	4.8 (5.2)	5.3 (5.1)	4.4 (4.6)	5.3 (5.2)	5.7 (4.7)	5.3 (4.9)	5.4 (4.8)	5.2 (4.9)
Joint pain *	5.7 (5.3)	6.7 (5.8)	4.7 (5.7)	7.4 (6.0)	5.9 (5.7)	5.2 (5.3)	7.1 (5.5)	6.1 (5.7)
Muscle aches	7.7 (5.1)	9.4 (5.6)	7.2 (5.2)	8.9 (5.8)	9.5 (5.3)	8.2 (5.3)	9.5 (5.3)	8.5 (5.4)
**DePaul Symptom Questionnaire (score range 0–16)**								
25—Muscle pain or ache *	7 (5.2)	8.4 (5.6)	6.5 (5.1)	8.3 (5.6)	8.4 (5.4)	6.8 (4.4)	9.1 (5.4)	7.7 (5.3)
26—Pain/stiffness without swelling >1 joint *	5.8 (5.6)	7.1 (6)	4.9 (5.4)	7.6 (6)	6.9 (5.7)	5.5 (5.1)	7.2 (5.4)	6.4 (5.6)
27—Eye pain	2.3 (3.5)	2.2 (3.6)	1.7 (2.7)	2.2 (3.5)	2.6 (4.1)	1.9 (3)	2.1 (3.7)	2.1 (3.4)
28—Chest pain	1.8 (3.6)	0.9 (2.3)	2.2 (3.7)	2 (3.5)	1.5 (2.2)	1.4 (2.6)	1.2 (2.4)	1.6 (3.0)
29—Bloating	4 (4.3)	4.8 (5.1)	3.8 (4.8)	4.1 (4.6)	3.6 (4)	2.9 (4)	3.4 (4.6)	3.8 (4.5)
30—Stomach pain	2.5 (2.8)	3.2 (4.1)	3.1 (3.7)	3.4 (4.6)	3.5 (4.2)	2.7 (3.1)	2.5 (3.4)	2.9 (3.7)
31—Headaches	4.4 (4.7)	4.6 (4.7)	4.3 (3.9)	4.6 (4.2)	4.5 (3.7)	4.1 (3.6)	4.7 (4.8)	4.4 (4.2)

All values are mean (standard deviation). *** *p* < 0.001; ** *p* < 0.01; * *p* < 0.05.

**Table 10:** Measures of other symptoms by site (A through G) and overall.

	A (n = 71, 15.3%)	B (n = 50, 10.8%)	C (n = 72, 15.5%)	D (n = 75, 16.1%)	E (n = 46, 9.9%)	F (n = 74, 15.9%)	G (n = 77, 16.7%)	Overall (n = 465)
**Immunologic/inflammation**								
CDC SI (score range 0–16)								
Sore throat	1.7 (3.0)	2.8 (3.9)	1.8 (3.1)	2.4 (3.5)	1.9 (3.0)	1.4 (2.7)	2.7 (3.7)	2.1 (3.3)
Sinus problems	6.0 (5.6)	4.9 (5.6)	3.7 (4.6)	4.8 (5.4)	4.5 (5.1)	3.8 (4.7)	5.0 (5.4)	4.7 (5.2)
Tender lymph nodes *	4.9 (5.6)	4.5 (4.8)	2.4 (3.8)	4.5 (4.6)	4.6 (5.3)	3.2 (4.2)	3.2 (4.6)	3.8 (4.8)
Fever	1.2 (2.3)	0.8 (1.9)	1.0 (2.5)	0.6 (1.6)	0.9 (1.7)	0.6 (1.3)	0.7 (1.6)	0.8 (1.9)
Chills	1.9 (3.2)	1.8 (3.1)	1.9 (3.1)	2.4 (3.6)	1.8 (3.7)	1.8 (3.1)	2.3 (3.8)	2.0 (3.4)
**DePaul Symptom Questionnaire (score range 0–16)**								
62—Sore throat	2.5 (3.3)	2.6 (3.5)	2.5 (3)	2.7 (3.4)	2.8 (3.3)	2 (2.3)	2.7 (3.3)	2.5 (3.2)
63—Tender lymph nodes	4.1 (4.3)	4 (4.6)	2.6 (3.3)	4.4 (4.4)	4.7 (4.9)	3 (3.9)	3.3 (4.3)	3.7 (4.2)
64—Fever **	1.5 (2.5)	1.1 (1.8)	1.4 (2.3)	0.6 (0.9)	1.7 (3)	0.6 (1.1)	0.8 (1.7)	1.1 (2.0)
65—Flu-like symptoms	4.7 (4.5)	4 (4.9)	5.2 (4.8)	4.8 (4.7)	5.7 (4.8)	4.4 (4.1)	3.5 (4.4)	4.6 (4.6)
66—Sickened by smells, food, medications, chemicals	4.8 (5)	5.2 (5.8)	4.2 (5.3)	4.2 (4.9)	6.5 (6)	4.3 (5)	5 (5.1)	4.8 (5.2)
**Gastrointestinal**								
CDC SI (score range 0–16)								
Stomach pain	2.8 (4.1)	2.3 (4.0)	2.9 (4.4)	3.5 (4.8)	2.9 (3.8)	3.2 (4.3)	3.4 (4.9)	3.0 (4.4)
Diarrhea	1.1 (2.3)	1.8 (3.3)	0.9 (2.5)	1.8 (3.3)	1.9 (3.2)	1.5 (3.4)	1.9 (3.4)	1.5 (3.1)
**Emotional or behavioral**								
CDC SI (score range 0–16)								
Depression	1.5 (3.4)	1.5 (2.8)	2.4 (3.9)	3.1 (4.8)	3.3 (4.1)	2.7 (4.1)	2.4 (4.1)	2.4 (4.0)
**Zung SDS (score range 0–80)**								
	42.6 (7.7)	42.8 (8.4)	46.0 (9.8)	46.7 (9.7)	45.6 (10.4)	45.3 (8.8)	45.3 (8.1)	45.0 (9.0)
**GAD-7 Anxiety (score range 0–21)**								
	3.9 (5.2)	5.0 (5.4)	5.3 (5.4)	6.7 (6.2)	4.7 (4.1)	4.7 (5.1)	4.7 (5.2)	5.1 (5.3)
**CDC Health-related QoL (0–30 days)**								
Mentally unhealthy days	8.3 (10.2)	9.0 (9.4)	10.7 (11)	8.4 (11.5)	10.6 (10.6)	10.1 (10)	7.8 (10.1)	9.2 (10.5)
**PHQ-8 Depression (score range 0–24)**								
	9.8 (5.4)	9.6 (5.8)	10.2 (5.5)	11.4 (5.1)	9.0 (4.9)	9.7 (5)	10.0 (4.1)	10.0 (5.1)

All values are mean (standard deviation). *** *p* < 0.001; ** *p* < 0.01; * *p* < 0.05.

**Table 11:** Patients meeting case definition algorithms by site (A through G) and overall.

Case Definition Number Meeting (%)	A (n = 71, 15.3%)	B (n = 50, 10.8%)	C (n = 72, 15.5%)	D (n = 75, 16.1%)	E (n = 46, 9.9%)	F (n = 74, 15.9%)	G (n = 77, 16.7%)	Overall (n = 465)
1994 Research	58 (81.7%)	45 (90.0%)	58 (80.6%)	64 (85.3%)	39 (84.8%)	57 (77.0%)	67 (87.0%)	388 (83.4%)
2003 Canadian	33 (46.5%)	24 (48.0%)	35 (48.6%)	41 (54.7%)	22 (47.8%)	34 (45.9%)	44 (57.4%)	233 (50.1%)
2015 IOM	38 (53.5%)	26 (52.0%)	43 (59.7%)	42 (56.0%)	29 (63.0%)	44 (59.5%)	46 (59.7%)	268 (57.6%)

## Data Availability

Restrictions by the data custodians mean that the datasets are not publicly available or able to be provided by the authors. The program codes used in the current study are available from the corresponding author on reasonable request. Researchers wanting to access the datasets used in this study should email CDC’s ME/CFS Program (cfs@cdc.gov) and discuss next steps for the data request. The ME/CFS program data review committee will grant the access after the review and the data use agreement is finalized.

## Supplementary Materials

The following supporting information can be downloaded at: https://www.mdpi.com/article/10.3390/jcm13051369/s1, Supplement S1. Practice Information Form-Characteristics of Clinicians and their Practices. Supplement S2. Operationalize three case definitions for ME/CFS.
