# Supplementary material for: Heterogeneity in Measures of Illness among Patients with Myalgic Encephalomyelitis/Chronic Fatigue Syndrome Is Not Explained by Clinical Practice: A Study in Seven U.S. Specialty Clinics
Source: J Clin Med. 2024 Feb 28;13(5):1369. doi: 10.3390/jcm13051369 (PMC10931716; doi:10.3390/jcm13051369)
Supplement: Supplementary file 1 [file jcm-13-01369-s001.zip › jcm-2803305-supplementary.pdf]

Supplement S1
Practice Information Form - Characteristics of Clinicians and their Practices

In the following questions, we would like to know more about you, your team and clinical practice. By "clinical practice," we mean all the clinics where you have been practicing medicine over the past year.

Part 1: Practice Structure

1. In which of the following US geographic regions is your practice located?
    a. Northeast
    b. Midwest
    c. South
    d. West

2. Which of the following areas best describes your practice location?
    a. Metropolitan
    b. Urban
    c. Rural

3. What types of transportation are accessible to your patients and their caregivers? Mark all that apply.
    a. Public transportation
    b. Hospital or clinic shuttle
    c. Free or validated parking
    d. Other (Specify:_________________)

4. Which of the following best describes your practice type?
    a. Solo practice **–> (Skip to Question 6)**
    b. Group practice
    c. Hospital-based practice
    d. Employed physician practice
    e. Other (Specify:_________________)

5. If you are not in a solo practice, how many **other physicians** are associated with the practice where you see the most patients?
   __ __ __ PHYSICIANS

6. Which of the following specialties are provided by the practice? Mark all that apply.
    a. General and family practice
    b. Internal medicine
    c. Neurology
    d. Psychiatry
    e. Other (Specify:_____________________)

7. Have you ever referred patients to other healthcare professionals outside of your practice?
    a. No

b. Yes, specify types of specialist/healthcare professionals
____

8. How long has the practice been in operation?
__ __ YEARS __ __ MONTHS

9. Over the past 5 years, which of the following healthcare models are provided by the practice? Mark all that apply.
    a. Multi-specialty care
    b. Compassion care
    c. Individualized medicine
    d. Precision Medicine
    e. Integrated care
    f. Integrative care
    g. Other (Specify:_____________________)

Part 2: Team Structure

10. Please specify the total numbers of full time equivalent (FTE) physicians and clinical staff in your practice: __ __ __ FTEs per year

    [Example of Calculation of FTE: **In a typical week,**
    if your practice has 1 physician working 20 hours/week, 1 nurse practitioner working 20 hours/week, 2 nurses working 20 hours/week, and 1 clinical exercise physiologist working 20 hours/week, the FTEs of your practice per year is calculated as follows: {(20x1) + (20x1) + (20x2) + (20x1)}/40= 2.5 FTEs]

11. The questions in the following table ask demographic information about each member in your practice (abbreviations: Doc= Doctor/Physician; PA= Physician Assistant; NP= Nurse Practitioner)

	Age Group (18-44, 45-64, 65-74, >=75)	Sex (F/M)	Race/Ethnicity (White; Black/African American; American Indian; Asian/Pacific Islander; Hispanic or non-Hispanic)	Years of practicing medicine (<5, 5-9, 10-14, 15-19, >=20)	Countries of medical education or training	Subspecialty (Pain, Sleep, Fibromyalgia, etc.)
Doc 1	45 – 64	F	White, Non-Hispanic	10 – 14	U.S.	Pain
Doc 2	>= 75	M	Asian/Pacific Islander, Hispanic	>20	Mexico	Sleep
Doc 1						
Doc 2						
Doc 3						
PA 1						
PA 2						
NP 1						
NP 2						
Nurse 1						
Nurse 2						

Nurse 3						

Part 3: Service and Cost

12. Does the practice currently accept new patients?
    a. Yes
    b. No

13. How many patients are currently on the waiting list to be seen in the practice? __ __ __PATIENTS

14. In a typical year, about how many weeks does your practice NOT see any patients (e.g., conferences, vacations, etc)?
    __ __ WEEKS

15. **During your last normal week of practice**, how many patients were seen IN THE PRACTICE?
    __ __ __ PATIENTS PER WEEK

16. On average, how much time (in minutes) is spent with a patient during the following type of visits:
    a. Initial visit: __ __ __ MINUTES
    b. Follow-up visit: __ __ __ MINUTES

17. What does your practice charge for care? **(Includes charge before discount.)**
    a. Initial visit _ _ _ _ _ (DOLLARS)
    b. Follow-up visit _ _ _ _ _ (DOLLARS)

18. Does your practice accept any insurance?
    a. Yes
    b. No **–> (Skip to Question 20)**

19. Please circle which type of insurance your practice accepts (circle all that apply)
    a. Medicare
    b. Medicaid
    c. Military (TRICARE, VA, CHAMP-VA)
    d. Private insurance
    e. Other (Specify:_________________)

20. Does your practice accept credit card or other payment plans for the office visit (i.e. co-pay or the whole charge)?
    a. Yes
    b. No

21. Does your practice use an electronic health record (EHR) system or electronic medical record (EMR)? **(Do not include billing record systems.)**
    a. Yes, all electronic
    b. Yes, part paper and part electronic } (**Go to Question 22)**
    c. No
    d. Unknown } (**Skip to Question 27)**
22. In which year did you install your current EHR/EMR system? __ __ __ __ Year

23. Does your current system meet meaningful use criteria as defined by the Department of Health and Human Services?
    a. Yes
    b. No
    c. Unknown

24. What is the name of your current EHR/EMR system?
    a. Cerner
    b. Epic
    c. Allscripts
    d. NextGen
    e. Other (Specify:_________________)

25. Has your practice made an assessment of the potential risks and vulnerabilities of your electronic health information within the last 12 months? This would help identify privacy or security related issues that may need to be corrected.
    a. Yes
    b. No
    c. Unknown

26. Does your EHR have the capability to electronically send health information to another provider whose EHR system is different from your system?
    a. Yes
    b. No
    c. Unknown

27. At your practice location, are there plans for installing a new EHR/EMR system within the next 18 months?
    a. Yes
    b. No
    c. Maybe
    d. Unknown

~The End of the Form~

Supplement S2

Operationalize three case definitions for ME/CFS:

A. **1994 Case Definition, operationalized algorithm**: meeting the following three criteria
    a. Fatigue criterion via MFI-20: General Fatigue > 13 OR Reduced Activity > 10, AND;
    b. Function impairment criterion via 0-100 transformed scores of SF-36: Physical Functioning < 70 OR Role Physical < 50 OR Social Functioning < 75 OR Role Emotional < 66.67, AND;
    c. Case-defining symptom criterion via CDC-SI: Case Definition Symptoms Score (from CDC Symptom Inventory) >= 25 AND Number of Symptoms > 4.

B. **Canadian Case Definition, operationalized algorithm** [Jason et al. The development of a revised Canadian myalgic encephalomyelitis chronic fatigue syndrome case definition. American Journal of Biochemistry and Biotechnology 6(2): 120-135, 2010.]: meeting all six categories below
    a. **Category 1 – Fatigue**: persistent or recurring chronic fatigue over the past six months that is not life-long and results in substantial reductions in previous levels of occupational, educational, social and personal activities.
    - Meet **all three criteria** below:
        - Not lifelong fatigue
            - Question-67 = “No”
        - Fatigue
            - Question-13 frequency >= 2 and severity >= 2
        - Substantial reduction in functioning: at least two of three following criteria
            - SF-36 Vitality <= 35
            - SF-36 Social functioning <= 62.5
            - SF-36 Role physical <= 50
    b. **Category 2 – Post-exertional malaise and/or post-exertional fatigue:** With activity, there must be a loss of physical or mental stamina, rapid/sudden muscle or cognitive fatigability, post-exertional malaise and/or post-exertional fatigue and a tendency for other associated symptoms within the patient’s cluster of symptoms to worsen.
    - Reported **one of** following five post-exertional malaise related questions/symptoms
        - Question-14 to Question-18: frequency >= 2 and severity >=2
    c. **Category 3 – Sleep**: Unrefreshing sleep, or disturbance of sleep quantity or rhythm.
    - Have **one of** following six sleep related questions/symptoms
        - Question-19 to Question-24: frequency >= 2 and severity >=2
    d. **Category 4 – Pain:** The person exhibits myofascial pain, joint pain, abdominal and/or head pain
    - Have **one of** following seven pain related questions/symptoms
        - Question-25 to Question-30: frequency >= 2 and severity >=2
        - Question-31 (headache): frequency >= 2 and severity >=2 and question-68 = “Yes”
    e. **Category 5 – Neurological/cognitive manifestations:**
    - Have **two or more** neurological/cognitive related questions/symptoms
        - Question-32 to Question-44: frequency >= 2 and severity >=2
    f. **Category 6 – Autonomic, neuroendocrine, or immune manifestations:**
    - Have **at least one symptom from two of** the three subcategories

- Autonomic manifestations
  - Question-45 to Question-51: frequency >= 2 and severity >=2

- Neuroendocrine manifestations
  - Question-52 to Question-61: frequency >= 2 and severity >=2
- Immune manifestations
  - Question-62 to Question-66: frequency >= 2 and severity >=2

Classification algorithm:

Classification = **Research ME/CFS**

a. Q69 (fatigue duration) = "6 – 12 months", "1 – 2 years", "longer than 2 years", or "Had problem with fatigue/energy since childhood or adolescence"

b. Meet all six categories above

C. 2015 IOM Case Definition, operationalized algorithm: meeting the following 4 criteria

a. Fatigue 6 month or longer and substantial reduction on occupational, educational, social or personal life (measured by CDC Symptom Inventory)
   - Fatigue 6 months and at least moderate for the severity level (i.e. a good bit of the time on frequency and moderate on intensity)
   - Not relieved by rest (When you are fatigued, does rest make your fatigue better? "No, not very much" or "No, not at all"
   - Substantially limited in any of occupational, educational, social or personal life

b. PEM 6 months or longer and at least moderate for the severity level (i.e. a good bit of the time on frequency and moderate on intensity, measured by CDC-SI)

c. Unrefreshing sleep 6 months or longer and at least moderate for the severity level (i.e. a good bit of the time on frequency and moderate on intensity, measured by CDC-SI)

d. OI or Cognitive: meeting one of the following two
   - Cognitive impairment for memory or concentration problem 6 months or longer and at least >=3 moderate for the severity level (i.e. a good bit of the time on frequency and moderate on intensity, measured by CDC-SI)
   - OI for dizziness 6 months or longer and at least moderate for the severity level (i.e. about half the time on frequency and moderate on intensity, measured by DSQ)
